# StemCellNet: an interactive platform for network-oriented investigations in stem cell biology

**DOI:** 10.1093/nar/gku455

**Published:** 2014-05-22

**Authors:** José P. Pinto, Ravi Kiran Reddy Kalathur, Rui S. R. Machado, Joana M. Xavier, José Bragança, Matthias E. Futschik

**Affiliations:** 1 Centre for Molecular and Structural Biomedicine, CBME/IBB, LA, University of Algarve, Faro, Algarve 8005-139, Portugal; 2 Department of Biomedical Sciences and Medicine, University of Algarve, Faro, Algarve 8005-139, Portugal; 3 Centre of Marine Science, University of Algarve, Faro, Algarve 8005-139, Portugal

## Abstract

Stem cells are characterized by their potential for self-renewal and their capacity to differentiate into mature cells. These two key features emerge through the interplay of various factors within complex molecular networks. To provide researchers with a dedicated tool to investigate these networks, we have developed StemCellNet, a versatile web server for interactive network analysis and visualization. It rapidly generates focused networks based on a large collection of physical and regulatory interactions identified in human and murine stem cells. The StemCellNet web-interface has various easy-to-use tools for selection and prioritization of network components, as well as for integration of expression data provided by the user. As a unique feature, the networks generated can be screened against a compendium of stemness-associated genes. StemCellNet can also indicate novel candidate genes by evaluating their connectivity patterns. Finally, an optional dataset of generic interactions, which provides large coverage of the human and mouse proteome, extends the versatility of StemCellNet to other biomedical research areas in which stem cells play important roles, such as in degenerative diseases or cancer. The StemCellNet web server is freely accessible at http://stemcellnet.sysbiolab.eu.

## INTRODUCTION

In recent years, numerous scientific breakthroughs have expanded our knowledge of stem cell biology, and inspired intense research efforts in this field. Aside from the evident importance of stem cells in developmental processes, their potential for clinical and medical applications has attracted much interest. Specifically, Yamanaka's invention of induced pluripotent stem cells (iPSCs) based on expression of exogenous transcription factors opened new avenues in patient-specific regenerative medicine (1). Their clinical use might be further developed by replacing current protocols with specific drug cocktails or with external stimuli (2,3). Furthermore, there is mounting evidence that stem cells (or cells with stem cell-like properties) play key roles in diseases such as cancer and degenerative disorders (4,5). For instance, malignant stem cells might be the culprits in various types of cancers, and be responsible for the frequently observed drug resistance and disease relapses (6,7). 

To probe the key features of stem cells, two major lines of investigations have emerged: (i) the systematic determination of genes that establish the core properties of stem cells (i.e. the capacity for self-renewal and for generation of differentiated progeny); and (ii) the large-scale detection of molecular interactions within stem cells (8–10). Whereas the first line of research has delivered various part lists for ‘stemness’ (i.e. defining features of stem cells), the latter has provided us with an initial basis with which to derive causal models for the processes underlying stem cell biology.

Although such research efforts have resulted in a considerable amount of data, their exploration and exploitation are restricted by the lack of dedicated computational resources, especially for researchers less acquainted with bioinformatics and computational biology. Such resources need to be comprehensive and flexible. They need to provide a high coverage of available interactions, while allowing for integration with other data types. Moreover, they should include tools for assessing the relevance of network components in order to help researchers prioritize further investigations.

StemCellNet provides this versatile platform. It is a web server for retrieval, and interactive analysis of molecular networks associated with stem cells and their marker genes. In particular, it is designed for rapid detection of a stemness signature in networks and for the identification of novel stem cell relevant genes, not only in stem cell biology, but also in other areas of biological and medical research.

## DATA INTEGRATED IN STEMCELLNET

Different types of data and information have been integrated in StemCellNet to enhance its versatility. In particular, StemCellNet combines both gene signatures for stemness, as well as stem cell specific interactions from numerous individual publications. Since stemness signature can depend strongly on the method used for their derivation (11,12), we provide a broad coverage of stemness signatures from different methods. Thus, we have curated over 20 distinct gene sets that are linked to stemness in previously published studies. These gene sets have been derived from three different types of genome-wide studies: (i) ChIP-chip experiments that detected activated target genes of the core transcription factors Nanog, Pou5f1 (Oct4) and Sox2 targets, or co-activation of all three transcription factors (referred as NOS targets) (13); (ii) gene expression studies that identified up-regulated genes in stem cells compared with other cell types (8,11,14–17); and (iii) large-scale functional RNAi screens that detected genes whose knock-down led to loss of stem cell markers (9,18–20).

Furthermore, a large number of regulatory and physical interactions, specifically identified in embryonic stem cells, were manually extracted from individual studies and integrated into StemCellNet. These comprise over 100 000 transcriptional regulatory interactions identified by the binding sites for key transcription factors using chromatin immunoprecipitation coupled with microarray technology (ChIP-chip) or ultra-high-throughput DNA sequencing (ChIP-Seq). Additionally, we collated almost 1000 physical protein interactions detected in embryonic stem cells using affinity purification and mass spectrometry for selected target proteins. Since these stem-cell specific interactions were derived mainly for a focused set of proteins with a known role in pluripotency, they cover only a small part of the human or murine proteome. To provide a more comprehensive coverage, we therefore also included molecular interactions for both human and mouse, identified in other types of cells. These additional molecular interactions (∼300 000) were imported from different resources (21,22). This expanded dataset enables the user to query interaction data for 36 023 distinct genes or proteins. Hence, StemCellNet can be used to scrutinize a large range of molecular processes. Notably, because StemCellNet tracks the source of each interaction, it allows users to easily trace these interactions back to the original publications, providing useful information for critical evaluation of the results. As described later, filtering options are available to exclude complementary generic interactions, i.e. interactions not specific to stem cells.

Finally, we integrated several transcriptomic and proteomic datasets that serve as a reference for network analyses. Among these, time-series for murine ESC differentiation within embryoid bodies, and generation of iPSCs are included (16,23). A detailed listing of all the data sets integrated in StemCellNet, as well as a description of our data processing can be found in the Supplementary Materials and on the StemCellNet Statistics page.

## STEMCELLNET WORKFLOW

**Figure 1.**
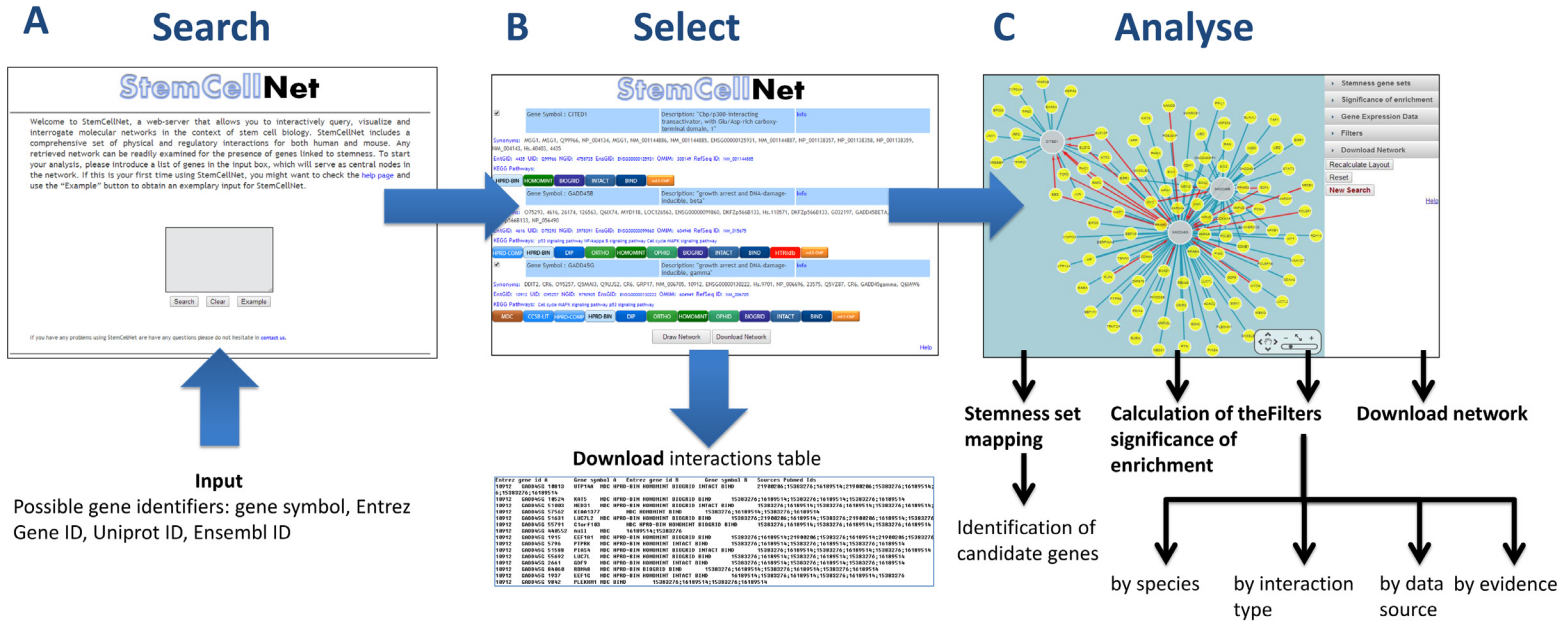
StemCellNet workflow. (**A**) Search: The user inputs a list of gene identifiers. (**B**) Selection: StemCellNet presents a list of genes contained in the associated database which match these identifiers. The user selects those that will serve as central nodes for the network. It is also possible to download a table containing all interactions for the matched genes. (**C**) Analysis: After selecting the central nodes, the network is generated and can be explored through integrated analysis tools.

The intention of StemCellNet is to provide an easy-to-use platform for network-orientated analyses in stem cell biology, as well as in other research areas, in which stem cells may play a role. Thus, the streamlined workflow consists of a sequence of three basic steps: *Search—Select—Analyse* (Figure 1). On the StemCellNet Home page, users can input identifiers for one or more human or mouse genes, which will serve as central nodes in the molecular interaction network. Currently, StemCellNet supports gene symbols, Entrez Gene IDs (human and mouse), as well as Uniprot IDs and Ensembl IDs (human only) as valid identifiers. These identifiers are then matched against genes and proteins for all interactions integrated in StemCellNet.

Next, all matches are presented to the user for selection and refinement. All source information for interactions, as well as all available annotation information is provided for each of the matched genes or proteins. Here, any undesired matches, due to the use of ambiguous gene symbols, can be excluded from analysis. To facilitate seamless integration of human and mouse data, and to avoid apparent duplication, orthologs of both organisms are automatically matched and only the human orthologs are presented. For the mapping of orthologs between mouse and human, gene annotations from the HUGO Gene Nomenclature Committee (HGNC) and the Mouse Genome Database (MGD) were used (24,25). We extracted the MGD ID (i.e. the mouse orthologs) for each human gene from the HGNC, and used these IDs to cross-index mouse genes with MGD annotations. If a mouse gene was associated with multiple human orthologs in the HGNC, it was mapped to all of these listed orthologs.

After selection of one or multiple genes by the user, all interactions of these genes and their corresponding proteins are retrieved through querying the relevant tables in the database. A detailed description of this database and its scheme can be found in the Supplementary Materials*.* The complete set of interactions found within StemCellNet can be exported as a simple table, which can be used by other software packages. This table also provides the original sources of interactions and their associated publications.

Alternatively, molecular interactions retrieved for the selected genes or proteins can be visualized and analysed as networks using StemCellNet. These networks are constructed by integrating all interactions found for the central nodes in the database. Here, the queried and selected genes define the set of central nodes in the network; only interactions with these genes are displayed in the current default view. Genes or proteins interacting with the central nodes are shown as connected nodes. Graphical network rendering is achieved through the Cytoscape Web application (26). For efficient interactive network visualization and analysis, it is advisable to limit the number of interaction partners to several hundred, as an excessive number of interactions will reduce the performance of the web server. This is most easily achieved by restricting the number of proteins that serve as central nodes. Thus, we recommend keeping the number of central nodes (i.e. the selected genes) small. Typically, network visualization involves up to 20 central nodes. Inclusion of interaction-rich genes or proteins, however, will necessitate a reduction in the number of nodes. Additionally, StemCellNet invokes an automatic layout and filtering procedure for the visualization of larger networks, and restricts the display of transcription regulatory interactions to the incoming type, i.e. regulatory interactions, which act on a target gene (see Supplementary Materials). The restriction of the graphical display can be circumvented using the direct download option, as pointed out above. Choosing this option, interactions for the full set of queried genes can be obtained.

Central nodes of the network can also be specified explicitly in the HTML address (i.e. by deep links) of StemCellNet. This feature can be exploited in other databases or web servers to offer their users the network context for specific genes provided by StemCellNet. It may also facilitate the use of StemCellNet in collaborative research. The Supplementary Materials include a number of examples of the syntax required for this purpose.

## NETWORK VISUALIZATION AND ANALYSIS

Network visualization and analysis constitute the central user interface in StemCellNet. Here, the generated molecular networks are displayed, showing both physical and regulatory interactions. All components (i.e. genes and proteins, as well as their interactions) can be interactively explored for additional information about their source, evidence, type and functional annotation. An integrated menu offers a variety of functions to examine the network, as described below. A step-by-step tutorial is available on the StemCellNet Help page.

### Screening for stemness signatures

Stemness signatures can help to rapidly identify genes involved in stem cell function within complex networks. They promise to be powerful tools in medical applications such as cancer prognosis, where their appearance in gene expression data correlates well with the clinical grading of various tumour types and also is significantly associated with a poor clinical outcome (4,14,27–29). StemCellNet screens networks for the presence of genes that have previously been associated with stemness. At present, we have included over 20 published stemness signatures (defined by transcription factor binding, gene expression studies, or the outcome of genome-wide RNAi screens), which can be user-selected in the menu. In addition to the stemness signatures derived by genome-wide experiments, sets of genes linked to stem cell maintenance and differentiation in gene ontology are available as further reference sets.

**Figure 2.**
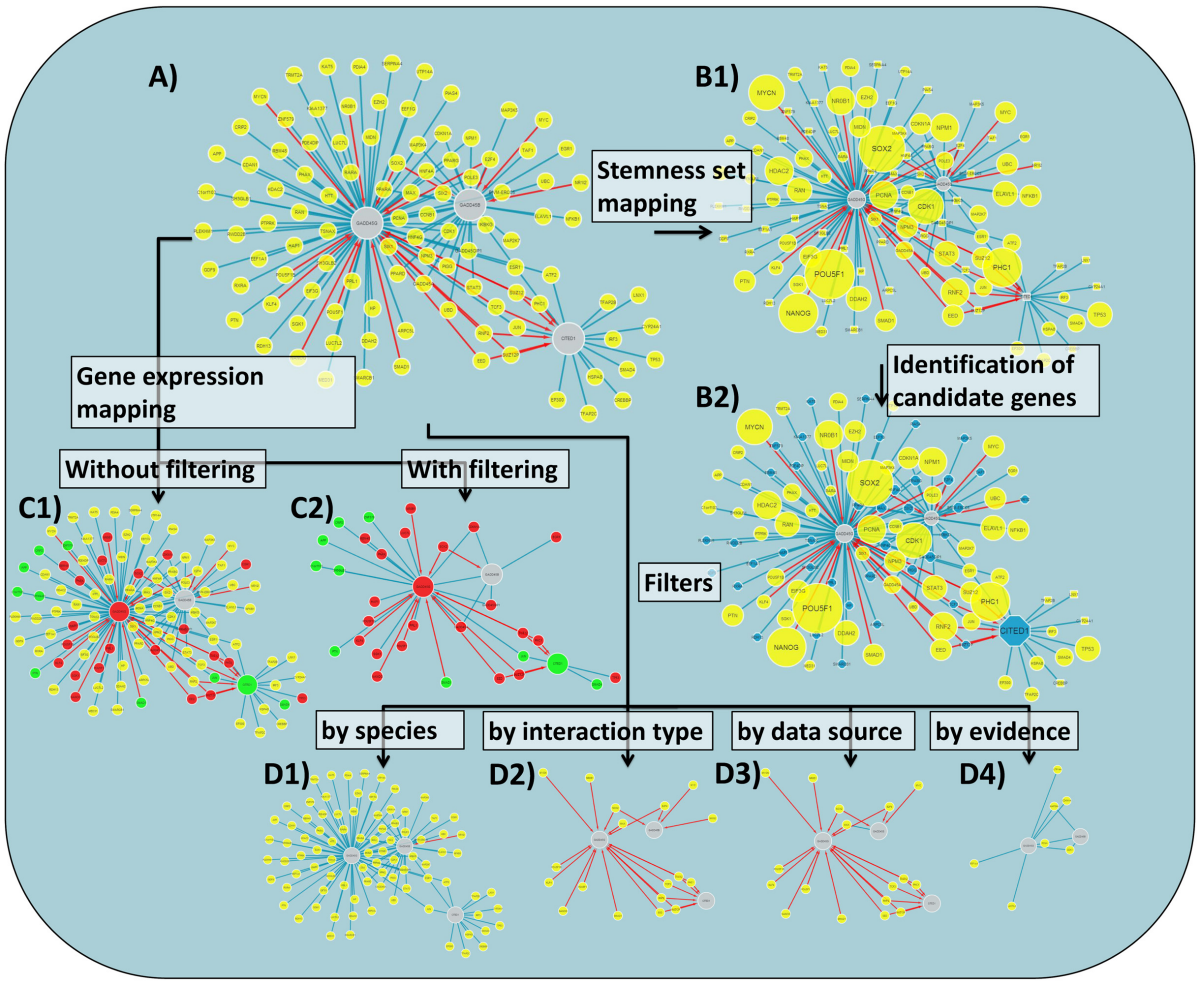
Network visualization and analysis in StemCellNet. (**A**) Default rendering of network for Cited1, Gadd45b and Gadd45g as exemplary input. The central proteins are represented by large grey nodes, while the interactors are represented by yellow nodes. Physical protein interactions are represented by blue undirected edges, while regulatory interactions are represented by red directed edges. (**B**) Stemness screen: (B1) Stemness association: Nodes are resized according to the number of gene sets to which the corresponding gene belongs; (B2) Identification of candidate nodes: Nodes that have not been associated with stemness gene sets are indicated as potential novel candidates by blue octagons and resized according to their connectivity to stemness genes within the displayed network. (**C**) Gene Expression integration: (C1) Red and green are used to highlight nodes whose corresponding gene or protein is differentially expressed based on a user defined cut off value, whereas nodes with non-differential expression retain their default colour (yellow or grey); (C2) Filtering of nodes by differential expression: Nodes are removed that are not differentially expressed, with the exception of central nodes. (**D**) Filtering of interactions: (D1) Filter by species: Edges are removed which were not found for a specific genome (i.e. human or mouse); (D2) Filter by interaction type: This function removes all physical or regulatory interactions from the network. (D3) Filter for stem cell specific interactions: Generic interactions are removed, leaving only interactions detected in stem cells. (D4) Filter by evidence: Interactions can be filtered by defining a minimal number of associated Pubmed IDs.

The results of the screen are graphically presented within the network view (Figure 2B1). In the rendered graph, the size of nodes is proportional to the number of stemness signatures, in which the corresponding genes are found. Nodes representing genes that do not appear in any stemness gene set are displayed with a distinct shape. In this way, genes re-occurring in many stemness signatures are emphasized, and can be easily pinpointed within the networks. The list of the gene sets, to which an individual gene belongs, can be seen in a pop-up window by clicking on the relevant node. The results of the screen can also be downloaded as table, in which genes are sorted according to their occurrence in stemness signatures. Additionally, their associated stemness gene sets are given.

### Significance of enrichment in stemness genes

As a complement to the gene-specific screen, the full network can be evaluated for enrichment in stemness-associated genes. Here, the statistical significance of an overrepresentation among the network components is calculated for each stemness gene set using Fisher's exact test. As output, an exportable table is produced, showing how many stemness genes were found within the network, and the value of significance for each stemness signature. It is not necessary to first map the sets before calculating the significance, but it should be noted that the calculations are based only on the nodes currently displayed, and are not automatically updated if filters are applied. We expect that, aside from its use in stem cell biology, this tool can help to dissect pathogenetic processes, in which stem cells play key roles, such as in degenerative diseases and oncogenesis.

### Identification of novel candidate genes associated with stemness

In some cases, it will be of interest to identify novel candidates genes associated with stemness. To assist researchers in this gene prioritization problem, we have implemented a tool that evaluates the connectivity within the network, and scores potential candidates accordingly. Currently, two variations of the ‘direct neighbour counting’ approach are available, which have been successfully applied in various fields of network biology (30). Both are motivated on the ‘guilt by association’ principle and assume (in our context) that the more stemness genes that a candidate gene is directly connected to, the more likely it is itself associated with stemness. In the unweighted scoring version, the score of a candidate is simply the sum of direct interactions with stemness associated genes. Alternatively, each interaction can be weighted by how many times the interacting genes occur in a stemness signature. This tool takes into account which stemness signatures are currently selected. Thereby, it is possible to control the input for the scoring method. In addition, we have implemented two variants of the method that use the correlation of expression between interacting genes for prediction of new stemness genes. As output, candidate genes are highlighted visually (Figure 2B2) and can be downloaded in table format. Other more sophisticated methods for gene prioritization will be implemented in future versions of StemCellNet.

### Integration of expression data

Expression data are indispensable to assess the activity of specific components within molecular networks. Therefore, StemCellNet integrates several time-resolved expression data sets for stem cell differentiation and reprogramming. These can be used as a ready reference for network analysis. More importantly, users can upload their own data using a simple table. The format conveniently allows a simultaneous upload of data from several samples, so that gene expression data for more complex experiments (such as time series) can easily be analysed. In either case, the expression data are mapped onto the network and nodes are coloured based on a user-defined threshold for fold changes or *P*-values. The colour-coding follows standard convention, with red indicating over- and green indicating under-expression (Figure 2C). Furthermore, the expression data can be employed for filtering of network components. Here, a minimal threshold for fold changes or *P*-values must be set. This feature can be overlaid with the graphical results of the stemness screen, providing supplementary information on the activity of stemness-associated genes.

### Filters for interactions

Several filtering options for interactions can be employed in StemCellNet to obtain more specified or reliable networks (Figure 2D). These include:

- *Filter for stem cell specific interactions*: All generic interactions can be removed, leaving a network consisting only of interactions detected in stem cells.
- *Filter by species*: This option can be employed to derive species-specific networks for human or mouse.
- *Filter by interaction type*: This function removes either all physical or all regulatory interactions from the network.
- *Filter by co-expression*: By setting a threshold value for correlation of expression for interacting genes or proteins, co-expressed sub-networks can be derived.
- *Filter by evidence*: To obtain greater confidence in the generated networks, the user can specify that the displayed interactions are reported by multiple publications by setting a minimum number of associated Pubmed IDs.

To avoid cluttering of networks with orphan nodes, all nodes that are unconnected after filtering are removed. Note that filters can be applied sequentially, so that different filtering combinations can be explored. Finally, the width of edges can be selected to reflect the number of Pubmed IDs associated with an interaction or the strength of co-expression between interacting partners. In this manner, an information-rich visualization of the interaction network can be achieved.

## STEMCELLNET: A UNIQUE WEB-BASED RESOURCE

StemCellNet complements and extends the currently available repertoire of on-line tools for stem cell biology, some of which are also described in Supplementary Materials. In contrast to gene-centric resources, such as Amazonia, Gene Expression Commons and SyStemCell (17,31,32) that return accumulated data for individual genes, StemCellNet enables researchers to assemble networks for a set of genes. It offers enhanced interactive network display and analysis compared with the recently established ESCAPE database, which also is based on accumulated molecular interaction data from stem cells (33). As such, StemCellNet is a unique resource, combining molecular interaction data with a variety of stemness signatures for network-oriented investigations. Importantly, the optional inclusion of generic interactions provides a broad coverage of molecular processes, making it a powerful tool for researchers of different areas. For instance, we anticipate that researchers working on degenerative diseases and on cancer will find StemCellNet useful to identify the potential roles of stem cells or stemness-associated processes in their systems of interest. We would like to emphasize that the inclusion of generic interactions does not result in dilution of context specific information for stem cell biologists, since the user can readily exclude these interactions.

## IMPLEMENTATION

StemCellNet was written using a combination of JavaScript and JavaServer Faces (JSF) 2.1, a Java-based framework for the development of user interfaces. The PrimeFaces library was used to expand functions available in JSF. Network visualization was facilitated through Cytoscape Web, a network browser designed for web applications (26). The database associated with StemCellNet was implemented using MySQL. A database scheme can be found in Supplementary Materials (Figure S1). The Hibernate library was employed to handle communications between StemCellNet and the database. Gene annotation was imported from UniHI database (21). Gene and protein identifiers used in the mapping function were uploaded from HGNC and bioDBnet resources (24,34). Generic interactions will be regularly updated as new versions of the original resources are released. Additional stem-cell specific interactions and stemness signatures will be integrated as they are identified in an ongoing literature review.

## FUTURE DIRECTIONS

StemCellNet is an active project. We will continue to expand its scope by adding new interaction data from genomic and proteomic studies of stem cells, as well as by implementing new analysis tools. Since we expect rapid growth of these data, StemCellNet provides documentary pages about the data content in each version. These pages will enable the user to easily assess the current state of the web server. Although we have concentrated on the curation of genomic and proteomic studies in the initial phase, we will also include data from small-scale studies in future versions of StemCellNet. To assist in this formidable task, we invite other researchers to submit their suggestions for the inclusion of studies or data sets in StemCellNet through dedicated web pages, and to assist with their curation. We hope that such features will transform StemCellNet into a community-based project.

Similar to the curation of stem cell-specific interaction datasets, the inclusion of published stemness signatures is an ongoing process. We also encourage other scientists to refer us to published signatures, which have not yet been included, for consideration in future versions of StemCellNet. Here, we are especially interested in adding signatures for defined sub-types of stem cells, such as for adult or malignant stem cells.

Besides enhancing our data coverage, we also seek to improve the performance of the web server. To date, only queries with a maximum number of 500 genes are allowed in StemCellNet to guarantee a rapid response time. When more genes are queried, the list is automatically truncated. Similarly, a maximum number of 20 genes can be selected as central nodes for visualization. We will attempt to relax these constraints, by optimizing both the database access and data processing in future versions of StemCellNet. We are also considering the possibility of developing a Cytoscape plug-in, capable of accessing the StemCellNet database to facilitate the analysis of larger networks. Additionally, we intend to implement an Application Programming Interface, so that other software applications can directly access StemCellNet to retrieve stem-cell specific interactions or even fully annotated networks.

## CONCLUSIONS

Stem cells have evoked considerable interest, not only due to their potential in regenerative medicine, but also because of their role in major diseases such as cancer. Recent experiments have begun to probe the molecular networks underlying stem cell maintenance and differentiation, and to define molecular signatures for stemness. To provide the research community with a tool that exploits the data generated in these lines of research, we have developed an interactive web server called StemCellNet. It is a much needed computational resource for the interactive analysis of molecular networks in stem cell biology, for which the number of dedicated software tools is still limited (17,31,33,35). It is the only current platform, which allows the screening of networks for stemness-associated genes and potential target candidates. Finally, with its comprehensive coverage of the human interactome, it is a powerful tool for researchers to use in order to identify stemness signatures in a wide range of biological processes. We believe that StemCellNet constitutes a unique web server, enabling biologists to tackle the inherent complexity of molecular networks associated with stem cells.

## SUPPLEMENTARY DATA

Supplementary Data are available at NAR Online.

Supplementary Data
