# Supplementary material for: StemCellNet: an interactive platform for network-oriented investigations in stem cell biology
Source: Nucleic Acids Res. 2014 May 22;42(Web Server issue):W154–60. doi: 10.1093/nar/gku455 (PMC4086070; doi:10.1093/nar/gku455)
Supplement: Supplementary Data [file supp_42_W1_W154__index.html]

Supplementary Data 

# StemCellNet: an interactive platform for network-oriented investigations in stem cell biology

## Supplementary Data

**Files in this Data Supplement:**

- SUPPLEMENTARY DATA
