# Supplementary material for: StemCellNet: an interactive platform for network-oriented investigations in stem cell biology
Source: Nucleic Acids Res. 2014 May 22;42(Web Server issue):W154–60. doi: 10.1093/nar/gku455 (PMC4086070; doi:10.1093/nar/gku455)
Supplement: Supplementary Data [file supp_gku455_nar-00281-web-b-2014-File004.pdf]

Supplementary Materials

StemCellNet: an interactive platform for network-oriented investigations in stem cell biology

José P. Pinto, Ravi K. Kalathur, Rui S. R. Machado, Joana M. Xavier, José Bragança and Matthias E. Futschik

Contents

1. Data collection, preprocessing and curation

1.1 Stemness signatures 2
1.2 Stem-cell specific physical protein interactions 2
1.3 Stem-cell specific transcriptional regulatory interactions 3
1.4 Generic interactions 3
1.5 Expression data sets 4
1.6. Current and future curation strategy 5

2. Automatic layout and filtering procedures for network visualization of large networks 7

3. Deep linking into StemCellNet 8

4. Database structure 9

5. Online resources for stem cell biology 10

5. Supplementary Table S1 11

6. Supplementary Table S2 11

7. Supplementary Table S3 12

8. Supplementary Table S4 13

9. Supplementary Table S5 14

10. Figure S1 15

11. References 16

1. Data collection, preprocessing and curation

Stemness signatures

We have included in StemCellNet gene signatures for stemness, i.e. gene sets linked to the capacity of stem cells to both self-renew and differentiate into mature cells. To collect the gene sets associated with stemness, we conducted a literature review and found gene lists related with stem cell signatures published in ten different studies performed in human (Boyer et al.,2005, Assou et al. 2007, Palmer et al. 2012) or mouse (Ivanova et al. 2012, Ramalho-Santos et al., 2002, Fortunel et al. 2003, Gaspar et al. 2012, Chia et al. 2010, Ding et al. 2009; Hu et al. 2009, Wang et al. 2012) samples.

Altogether, twenty distinct gene sets were derived from either ChIP-chip experiments detecting activated target genes of the core transcription factors Nanog, Pou5f1 (Oct4), and Sox2 targets, or co-activated by all three transcription factors (Boyer et al.,2005); gene expression studies to identify up-regulated genes in stem cells compared to other types of cells (Assou et al. 2007, Palmer et al. 2012, Ivanova et al. 2012, Ramalho-Santos et al., 2002, Fortunel et al. 2003, Gaspar et al. 2012) or large-scale functional RNAi screens to detect genes whose knock-down leads to loss of stem cell markers (Chia et al. 2010, Ding et al. 2009; Hu et al. 2009, Wang et al. 2012). See supplementary table S1 for an overview of the currently included stemness signatures.

Stem-cell specific physical protein interactions.

Stem-cell specific physical protein interactions (PPI) were extracted from eight studies (Wang et al., 2006; Liang *et al*., 2008; Kim et al., 2010; Pardo et al., 2010, van den Berg et al., 2010; Ding et al., 2012; Gao et al., 2012; Nitzsche et al., 2011) that applied either affinity purification or mass spectrometry methods against the selected proteins. All protein interactions identified and described in the published papers were uploaded to the StemCellNet by extracting the data from the published supplementary information. Proteins were identified by the corresponding Entrez Gene ID. Supplementary table S2

presents the sources and further details for the collected stem-cell specific physical protein interactions.

Stem-cell specific transcriptional regulatory interactions.

The data from either ChIP-chip, ChIP-PET or ChIP-Seq was extracted from eleven different studies [Chen et al., 2008, Kim et al., 2010, Marson et al., 2008, Kim et al., 2008, Cole et al., 2008, Liu et al., 2008, Loh et al., 2006, Boyer et al., 2006, Tam et al., 2008, Han et al., 2010, Mathur et al., 2008]. An overview can be found in supplementary table S3.

In the paper by Chen and colleagues [Chen et al., 2008], the binding affinity between transcription factor and target genes showed quantitative values and the authors did not point out any specific threshold. Therefore, we applied different cutoffs for each TF in order to keep as a stringent measure only the 25% top scoring genes for each transcription factor. The minimum binding affinities selected were 0.221, 0.365, 0.278, 0.291, 0.400, 0.968, 0.730, 0.976, 0.878, 0.900, 0.976, 0.964, 0.970 and 0.534 for Nanog, Pou5f1, Sox2, Smad1, Stat3, Klf4, Myc, Myc-n, Esrrb, Tcfcp2l1, Zfx, E2f1, Suz12 and Ctcf, respectively.

For the data extracted from the remaining ten studies [Kim et al., 2010, Marson et al., 2008, Kim et al., 2008, Cole et al., 2008, Liu et al., 2008, Loh et al., 2006, Boyer et al., 2006, Tam et al., 2008, Han et al., 2010, Mathur et al., 2008] we selected the probes defined as bound to the transcription factor by the authors in the published papers.

For all the studies and for each TF individually, we excluded probes considered not to have binding affinity to the transcription factor and then filtered the probes according to unique gene IDs in order to keep only one interaction for each pair of TF-gene in each study. Genes with no known Entrez Gene ID or probes corresponding to cloning artifacts withdrawn by NCBI were excluded in the input in StemCellNet.

Generic interactions

To increase the coverage of StemCellNet, we imported human molecular interaction from the Unified Human Interactome (UniHI) database (http://www.unihi.org) and

murine protein interaction from the BioGRID database (http://thebiogrid.org/). These were termed “generic interactions” to emphasize that they were not specifically detected in stem cells, but detected in other tissue, cell types or in vitro assays. Details about the data curation can be found on the web-pages of the UniHI and BioGRID databases. As both databases release new versions in an ongoing manner, we will regularly update the imported datasets in into StemCellNet. Since UniHI is a meta-database, which integrates various primary resources and databases for protein interactions, we keep also track of the original resources and refer to these in the StemCellNet. Table S4 gives an overview of the primary sources integrated in the UniHI database.

Expression data sets

Expression datasets obtained either by microarray analysis or high-resolution nano liquid chromatography-tandem mass spectrometry were derived from four different studies [Aiba et al., 2009; Uosaki et al., 2011; Gaspar *et al*., 2012; Hansson et al., 2012]. Supplementary table S5 lists the expression data sets currently accessible in StemCellNet.

The dataset published by Gaspar et al. 2012 profiled RNA expression in the first 10 days of *in vitro* differentiation of murine embryonic stem cells (mESCs). From the 45,101 probe sets represented on the Mouse 430 version 2 array, expression data of 30,526 gene associated transcripts were analysed after eliminating transcripts without annotation and of unknown origin, as well as hypothetical transcripts or proteins. Genes with repeated Entrez Gene Ids were also excluded, resulting in 20881 unique Gene id.

In the data published by Uosaki and colleagues [Uosaki *et al*., 2011] human induced pluripotent stem cells (hiPSc) were differentiated towards cardiomyocytes applying sequential administration of activin, bone morphogenetic protein 4 (BMP4), fibroblast growth factor 4 (FGF4) and Dickkopf 1 homolog (DKK1). The expression profiling was performed with Affymetrix Human Gene 1.0 ST arrays at day 0, 2, 5, 7, 9 and 11 during differentiation. The *CEL* files were downloaded from GEO (accession number

GSE28191) and were submitted to background correction, normalization and summarization of gene expression (*rma*), using the R package *Affy* [Gautier et al., 2004]. After elimination of repeated Entrez gene Ids, a total of 19889 genes were covered by the expression .

For the study from Aiba et al, 2009, we analysed three different lineages/time series obtained from: (1) Endodermal 5G6GR (D+) plated on gelatin coated 10cm plate with DMEM containing Dexametasone(Dx) (100nM) in presence of LIF and cultured to 5 days; (2) Clones of ES cells isolated from Trophoblast ZHBTc4(Tc+) treated with ROSA-TET system an Tet inducible system and cultured in regular medium within 18 ug/ml zeocin (3) Neural N2 obtained by plating and culturing ES cells as a monolayer in N2B27 for 1 day. The data matrix series were extracted from GEO (accession number GSE11523). The Ref_ID of the matrix series were matched with the data table header descriptions to obtain the entrez gene id. From the 25164 genes present in the initial matrix series, we obtained a final list of 24287 genes, after Ref_ID from the matrix series without corresponding gene ID or symbol were excluded. Expression data of the replicates were averaged and all time series were adjusted, so that the mean expression of a gene equals 0 and presented as log2 fold changes.

In the proteomic study from Hansson et al., 2012 the authors applied in-depth quantitative proteomics to monitor proteome changes during the course of reprogramming of fibroblasts to iPSCs (Hansson et al., 2012). The data was extracted from the supplementary files available in Cell Reports (http://download.cell.com/cell-reports/mmcs/journals/2211-1247/PIIS2211124712003695.mmc1.xlsx). After filtering and excluding proteins that did not match any Entrez Gene ID or did not have any gene name, we obtained 7409 out of the initial 7918 proteins present in the study.

Current and future curation strategy

The current focus lies on the curation of genomic and proteomic studies reporting molecular interaction in stem cells. For this purpose, we performed an extensive and systematic review reported by PubMed when querying with a defined set of terms. The reported publications were then examined whether they included newly generated

interaction data. For instance, to obtain a comprehensive set of ChIP-chip and ChIP-seq studies reporting mapping transcription factor binding sites, we performed a Pubmed search using as mandatory key-words "embryonic stem cell and genome" combined with non-mandatory key words such as "chip-chip, chip-seq, chromatin immunoprecipitation, target, regulatory, transcription factor, expression and pathway". The selection of key-words will be broadened in future version to e.g. include interaction data for types of stem cells other than embryonic. To enable the assessment of the current state of StemCellNet by the user, the web-server provides pages documenting the included data in the different versions.

For future versions of StemCellNet, we will start to curate small scale studies. To obtain assistance by other researchers and experts for this task, we will set up a dedicated webpages, where suggestions to include studies or data sets can be placed or even curation of data can be undertaken by external researchers. We hope that such features can eventually help to transform StemCellNet into a community-based project.

2. Automatic layout and filtering procedure for network visualization of large networks

For network visualization, we used the Cytoscape Web (Lopes et al., 2010). This software shows a good performance with small to medium-sized networks (including up to several hundred nodes and edges) but becomes increasingly slow in the case of larger networks. Thus, to avoid lengthy response times or even a stalling display, several automatic adjustments were taken for visualization of networks larger than certain thresholds.

If the number of nodes is greater than 400, the network is rendered using radial layout, which is faster compared to the default force directed layout and can support larger networks but if the number of edges in the network surpasses 1000, a filtering procedure is performed. In this case, StemCellNet will attempt to show only interactions that have more than one PubMed ID associated with it. This approach seeks to retain the interactions linked to greater evidence. In the case that there are still more than 1000 edges after this filtering step, the minimal number of required PubMed IDs will be raised until the number of edges in the network is smaller than 1000. In all situations the user is alerted when automatic filtering occurs.

As many transcription factors have a large number of target genes, which makes their visualization readily inefficient, we restricted the display of regulatory interactions to the incoming type. This means that only regulatory interactions are shown which act upon target genes included in the network that is composed by the central proteins and their physical interactors.

Finally, it is possible to download the full set of interactions from the StemCellNet central node selection page. This data can be used as input for alternative stand-alone software tools, such as Cytoscape and R/Bioconductor, which may be more advisable for efficient analysis of large interaction networks.

3. Deep linking into StemCellNet

StemCellNet can help the users to recreate previously obtained search results or networks without having to repeat the search process, through deep links. This links contain the data necessary to reconstruct either a search result or network in the format of link parameters.

As an example, to deep link into a concrete protein or gene identifier search the user can use a link as the following:

http://193.136.227.168:8282/stemcellnet/stemCellNetNetworkResult.jsf?prot1=CITED1&prot2=GADD45B&prot3=GADD45G

This link's parameters contain the gene symbols (CITED1, GADD45B, GADD45G) which were found in the initial search by identifier. StemCellNet uses these parameters to reconstruct the dynamic search result page by requesting those same proteins from the associated database.

For network construction and visualization, StemCellNet uses internal database IDs instead of general gene/protein identifiers but the deep link works similarly to described above. Given an example, if all the central nodes from the previous example are used to create a network, the deep link into it will be:

http://193.136.227.168:8282/stemcellnet/stemCellNetNetwork.jsf?c=3673&c=3792&c=8837

4. Database structure

The database underlying StemCellNet was developed using MySQL relational database management system. Altogether, the database consists of 18 different tables (Figure S1) storing different types of data and information. There are 7 core tables including *proteins*, *interaction_distribution, regulatory_interactions, interaction_properties, stem_gene_expression* and *screen,* that hold all the essential information relating to proteins, their interactions and expression. The *proteins* table houses extensive annotation of proteins with *prid* as primary key. Protein-protein interactions and regulatory interactions are present in *interaction_distribution* and *regulatory_interactions* tables, respectively. These two tables are linked to *proteins* tables via *prid* as foreign key. Information about source of interaction is placed *interaction_properties* table, which is linked to *interaction_distribution* table through i*ntdisid* which is the primary key. Stem cell specific gene expression is stored and retrieved from *stem_gene_expression*. Currently, there are over 600.000 expression values present in the StemCellNet. In addition to gene expression data, stemness gene sets are stored in *screen* table. Both the *stem_gene_expression* and *screen* tables are linked to *proteins* tables via *prid*, the primary key of the *proteins* table

5. Online resources for stem cell biology

Several online resources for stem cell biology have been established in recent years. Many of them are gene-centric i.e. their main functionality is to provide collected data and information for individual queried genes. This is contrasted by resources such as StemCellNet, which enables interactive analysis of genes of interest within a network context. In the following, some of the currently available online resources and their applicability are briefly described.

A typical example of a gene-centric resource is StemCellDB (http://stemcelldb.nih.gov) by the NIH, which enables the querying of expression data for individual genes. It includes data measured in-house by Affymetrix or Agilent array technology for 21 human ESC lines as well as eight iPSC lines derived by retroviral transduction of human fibroblasts. Only basic tools for data analysis and display are provided. A similar database based on curated expression data sets is constituted by Stemformatics (http://www.stemformatics.org), which includes several more advanced tools for data visualization. A resource, which can map the expression of genes onto defined models of stem cell differentiation, is Gene Expression Commens (https://gexc.stanford.edu). Finally, a gene-centric database, in which other types of data for various levels of regulation can be found (apart of gene expression data), is SyStemCell (http://lifecenter.sgst.cn/SyStemCell/).

These gene-centric resources are contrasted by network-orientated resources, such as the StemCellNet web-server described in the main manuscript. One of the first efforts in this direction is represented by the StemCellMatrix web-site (http://www.stemcellmatrix.org), an online compendium of a publication, in which PluriNet, a molecular network model for pluripotency was described. However, many features on the web-site do not seem to be functional anymore. Finally, ESCAPE (http://www.maayanlab.net/ESCAPE) is a database, which integrates published data for human and mouse embryonic stem cells and enables the derivation of networks for query proteins similarly to our StemCellNet web-server.

Table S1: Stemness gene sets. Abbreviations: ESC – embryonic stem cells, NS – neural stem cells, HSC – hematopoietic stem cells

Stemness gene set	Organism	Stem Cell Type	Type of Source	Number of genes	Pubmed ID
Hs ESC Nanog Targets Boyer	*Homo sapiens*	ES Cells	TF Binding	988	16153702
Hs ESC Oct4 Targets Boyer	*Homo sapiens*	ES Cells	TF Binding	290	16153702
Hs ESC Sox2 Targets Boyer	*Homo sapiens*	ES Cells	TF Binding	734	16153702
Hs ESC NOS Targets Boyer	*Homo sapiens*	ES Cells	TF Binding	179	16153702
Hs ESC Consensus Assou	*Homo sapiens*	ES Cells	Expression	379	17204602
Hs SC Palmer	*Homo sapiens*	Diverse	Expression	187	22909066
Mm ESC Ivanova	*Mus musculus*	ES Cells	Expression	967	12228721
Mm NSC Ivanova	*Mus musculus*	NS cells	Expression	840	12228721
Mm HSC Ivanova	*Mus musculus*	HS cells	Expression	712	12228721
Mm ESC Ramalho-Santos	*Mus musculus*	ES Cells	Expression	1141	12228720
Mm NSC Ramalho-Santos	*Mus musculus*	NS Cells	Expression	1538	12228720
Mm HSC Ramalho-Santos	*Mus musculus*	HS Cells	Expression	1231	12228720
Mm ESC Fortunel	*Mus musculus*	ES Cells	Expression	1066	14563990
Mm NSC Fortunel	*Mus musculus*	NS Cells	Expression	1101	14563990
Mm ESC Gaspar	*Mus musculus*	ES Cells	Expression	160	22420508
Hs ESC Chia	*Homo sapiens*	ES Cells	RNAi	518	20953172
Mm ESC Ding	*Mus musculus*	ES Cells	RNAi	145	19345177
Mm ESC Hu	*Mus musculus*	ES Cells	RNAi	123	19339689
Mm ESC Wang	*Mus musculus*	ES Cells	RNAi	89	22899353

Table S2: Stem cell specific protein interactions

Target proteins	Number of interaction partners	Publication	Pubmed ID	Logo in StemCellNet
Pou5f1, Sall4, Tcfcp2l1, Dax1, Esrrb	267	van den Berg et al. Cell Stem Cell. 2010	20362541	mES-PPI
Pou5f1	198	Ding et al. Cell Res. 2012	22083510	
Nanog	132	Nitzsche et al. PloS One 2011	21589869	
Pou5f1	93	Pardo et al. Cell Stem Cell. 2010	20362542	
Myc, Max, Dmap1, Tip60, Gcn5, E2F4	84	Kim et al. Cell 2010	20946988	
Sox2	71	Gao et al. J Biol Chem. 2012	22334693	
Nanog, Nac1, Zfp281, Dax1, Pou5f1	66	Wang et al. Nature 2006	17093407	
Nanog, Pou5f1	24	Liang J. et al Nat Cell Biol 2008	18454139	

Table S3: Summary of the number of transcription factor regulatory interactions data detected by chromatin immunoprecipitation combined with microarray and sequencing technologies (ChIP-chip, ChIP-PET or ChIP-Seq) in different studies. Altogether, StemCellNet has over 10500 distinct regulatory interactions detected from murine embryonic stem cells in 11 large-scale studies.

Transcription factor	Number of target genes	Publication	PubMed ID	Logo in StemCellNet
Nanog, Pou5f1, Sox2, Smad1, Stat3, Klf4, Myc, Mycn, Esrrb, Tcfcp2l1, Zfx, E2f1, Suz12, Ctcf	29476	Chen et al.,Cell 2008	18555785	mES-ChIP
Gcn5, Dmap1, E2f4, Myc, Max, Tip60	17422	Kim et al ,Cell 2010	20946988	
Nanog, Pou5f1, Sox2, Suz12, Tcf3	13867	Marson et al.,Cell 2008	18692474	
Klf4, Myc, Nacc1, Nanog, Nr0b1, Pou5f1, Sox2, Zfp281, Zfp42	12407	Kim et al.,Cell 2008	18358816	
Nanog, Pou5f1, Tcf3	6239	Cole et al., Genes Dev.,2008	18347094	
Klf4, Sox2, Myc	6168	Liu et al., Cell Res. 2008	19030024	
Nanog, Pou5f1	5070	Loh et al.,Nat. Genet. 2006	16518401	
Eed, Phc1, Rnf2, Suz12	4243	Boyer et al., Nature 2006	16625203	
Nanog, Pou5f1	2478	Mathur et al.,Genome Biol.,2008	18700969	
Tcf3	1445	Tam et al.,Stem Cells,2008	18467660	
Tbx3	1701	Han et al.,Nature,2010	20139965	
Nanog, Pou5f1	2478	Mathur et al.,Genome Biol.,2008	18700969	

Table S4: Resources for generic human protein interactions imported into StemCellNet via the UniHI database.

Resource	Number of interactions	Type of interaction	Method	Reference	Logo in StemCellNet
MDC-Y2H	3340	Physical protein	Y2H screen	Stelzl et al. 2005 Cell	MDC
CCSB	3741	Physical protein	Y2H screen + Import from other databases	Rual et al. 2005 Nature	CCSB-LIT<br>CCSB-Y2H
HPRD	65227	Physical protein	Literature curation	Prasad et al. 2009 NAR	HPRD-BIN<br>HPRD-COMP
BioGRID	124035	Physical protein	Literature curation	Chatr-Aryamontri, A et al. 2013 NAR	BIOGRID
BIND	19352	Physical protein	Literature curation	Isserlin, R et al. Database, 2011	BIND
DIP	2925	Physical protein	Literature curation	Salwinski et al. NAR 2004	DIP
IntAct	37629	Physical protein	Literature curation	Kerrien et al. NAR 2012	INTACT
Reactome	108867	Physical protein	Pathway curation	Croft et al. NAR 2011	REACTOME
COCIT	6580	Functional association	Computational prediction	Ramani et al. 2004 Genome Biology	COCIT
ORTHO	62863	Physical protein	Computational prediction	Lehner et al. 2004 Genome Biology	ORTHO
HOMOMINT	21863	Physical protein	Computational prediction + Literature curation	Persico et al. 2005 BMC Bioinformatics	HOMOMINT
OPHID	81677	Physical protein	Computational prediction	Brown et al. 2005 Bioinformatics	OPHID
TRANSFAC (public version)	1554	Regulatory transcriptional	Literature curation	Matys et al. 2006 NAR	TRANSFAC
miRTarBase	3565	Regulatory transcriptional	Literature curation	Hsu SD et al. 2011 NAR	miRTarBase
HTRIdb	2263	Regulatory transcriptional	Literature curation	Bovolenta et al. 2012 BMC Genomics	HTRIdb

Table S5. Expression data sets time series integrated in StemCellNet. In the table is shown the reference publications, organism in which the experiments were performed, time points in which the data was colected, type os stem cell studied, number of targets collected, experimental technique used to collect the data in the original publications, database from where the data was collected and pubmed ID from the original publication.

**Reference**	**Organism**	**Time points**	**Stem Cell type**	**Number of genes/ proteins**	**Platform**	**GEO/ Arrayexpress/ Supplement**	**Pubmed ID**
Agapios *et al.*, 2012	*Mus musculus*	8	ES Cells	20881 Genes	Affymetrix GeneChip Mouse Genome 430 2.0	E-TABM-672	22420508
Uosaki *et al.*, 2011	*Homo sapiens*	6	hiPS Cells	19889 Genes	Affymetrix Human Gene 1.0 ST Array [transcript (gene) version]	GSE28191	21876760
Aiba *et al.,* 2009	*Mus musculus*	5	ES Cells	24287 Genes	NIA Mouse 44K Microarray v3.0 (Whole Genome 60-mer Oligo) [Agilent 015087]	GSE11523	19112179
Hansson *et al.*, 2012	*Mus musculus*	18	MEF Cells	7409 Proteins	LC-MS/MS	Supplementary Table S1	23260666

Figure S1: Scheme of database underlying StemCellNet

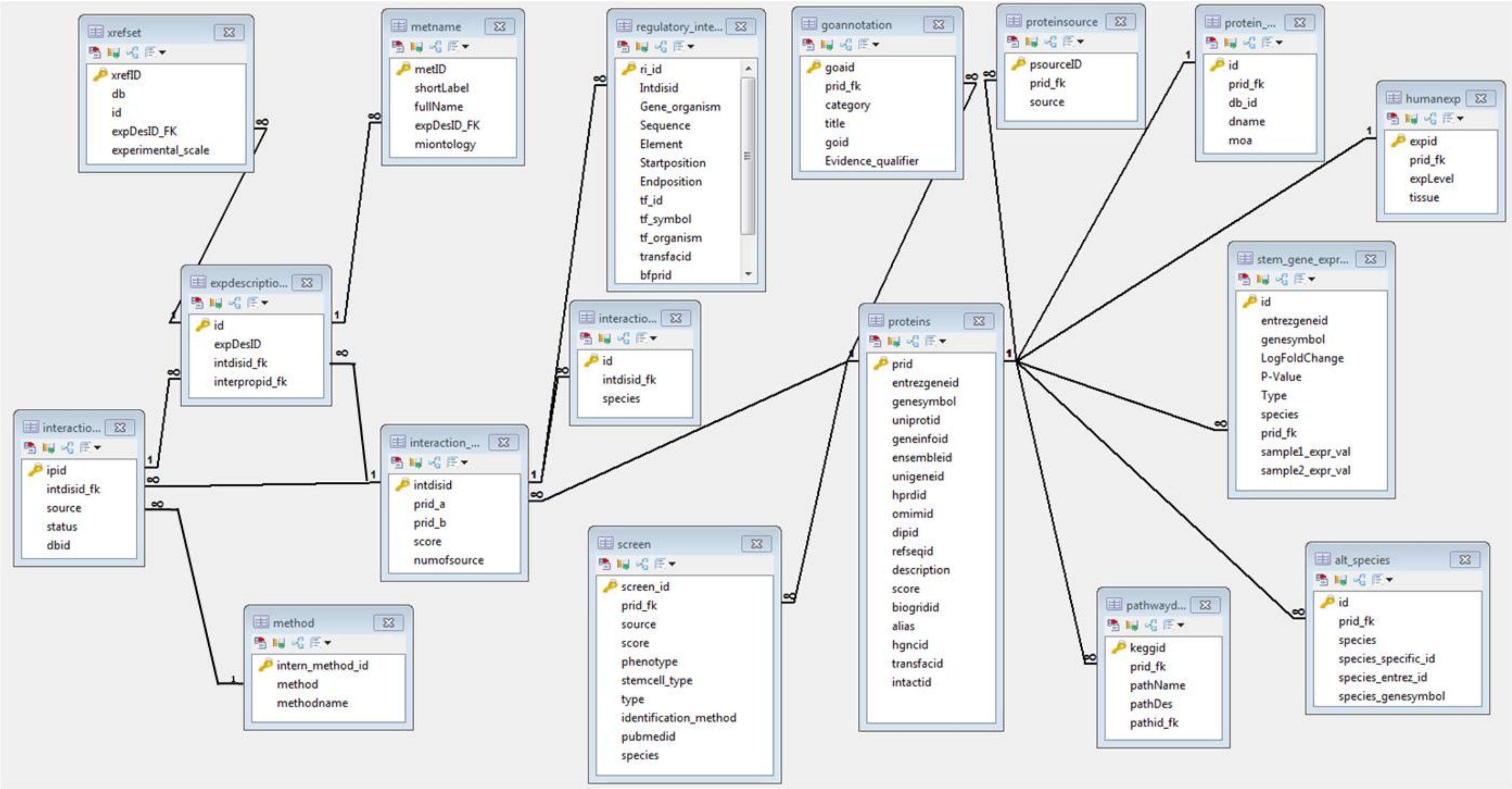

REFERENCES

Aiba, K., Nedorezov, T., Piao, Y., Nishiyama, A., Matoba, R., Sharova, L.V., Sharov, A.A., Yamanaka, S., Niwa, H., and Ko, M.S. (2009). Defining developmental potency and cell lineage trajectories by expression profiling of differentiating mouse embryonic stem cells. DNA research: an international journal for rapid publication of reports on genes and genomes, **16**, 73-80.

Assou, S., Le Carrour, T., Tondeur, S., Strom, S., Gabelle, A., Marty, S., Nadal, L., Pantesco, V., Reme, T., Hugnot, J.P. *et al.* (2007) A meta-analysis of human embryonic stem cells transcriptome integrated into a web-based expression atlas. *Stem cells (Dayton, Ohio)*, **25**, 961-973.

Bovolenta, L.A., Acencio, M.L., and Lemke, N. (2012). HTRIdb: an open-access database for experimentally verified human transcriptional regulation interactions. BMC genomics, **13**, 405.

Boyer, L.A., Lee, T.I., Cole, M.F., Johnstone, S.E., Levine, S.S., Zucker, J.P., Guenther, M.G., Kumar, R.M., Murray, H.L., Jenner, R.G. *et al.* (2005) Core transcriptional regulatory circuitry in human embryonic stem cells. *Cell*, **122**, 947-956.

Boyer, L.A., Plath, K., Zeitlinger, J., Brambrink, T., Medeiros, L.A., Lee, T.I., Levine, S.S., Wernig, M., Tajonar, A., Ray, M.K*., et al.* (2006). Polycomb complexes repress developmental regulators in murine embryonic stem cells. Nature, **441**, 349-353.

Brown, K.R., and Jurisica, I. (2005). Online predicted human interaction database. Bioinformatics, **21**, 2076-2082.

Chatr-Aryamontri, A., Breitkreutz, B.J., Heinicke, S., Boucher, L., Winter, A., Stark, C., Nixon, J., Ramage, L., Kolas, N., O'Donnell, L. *et al.* (2013) The BioGRID interaction database: 2013 update. *Nucleic acids research*, **41**, D816-823.

Chen, X., Xu, H., Yuan, P., Fang, F., Huss, M., Vega, V.B., Wong, E., Orlov, Y.L., Zhang, W., Jiang, J. *et al.* (2008) Integration of external signaling pathways with the core transcriptional network in embryonic stem cells. *Cell*, **133**, 1106-1117.

Chia, N.Y., Chan, Y.S., Feng, B., Lu, X., Orlov, Y.L., Moreau, D., Kumar, P., Yang, L., Jiang, J., Lau, M.S. *et al.* (2010) A genome-wide RNAi screen reveals determinants of human embryonic stem cell identity. *Nature*, **468**, 316-320.

Cole, M.F., Johnstone, S.E., Newman, J.J., Kagey, M.H., and Young, R.A. (2008). Tcf3 is an integral component of the core regulatory circuitry of embryonic stem cells. Genes & development, **22,** 746-755.

Croft, D., O'Kelly, G., Wu, G., Haw, R., Gillespie, M., Matthews, L., Caudy, M., Garapati, P., Gopinath, G., Jassal, B.*, et al.* (2011). Reactome: a database of reactions, pathways and biological processes. Nucleic acids research, **39**, D691-697.

Ding, L., Paszkowski-Rogacz, M., Nitzsche, A., Slabicki, M.M., Heninger, A.K., de Vries, I., Kittler, R., Junqueira, M., Shevchenko, A., Schulz, H. *et al.* (2009) A genome-scale RNAi screen for Oct4 modulators defines a role of the Paf1 complex for embryonic stem cell identity. *Cell stem cell*, **4**, 403-415.

Ding, J., Xu, H., Faiola, F., Ma'ayan, A., and Wang, J. (2012). Oct4 links multiple epigenetic pathways to the pluripotency network. Cell research, **22**, 155-167.

Fortunel, N.O., Otu, H.H., Ng, H.H., Chen, J., Mu, X., Chevassut, T., Li, X., Joseph, M., Bailey, C., Hatzfeld, J.A. *et al.* (2003) Comment on " 'Stemness': transcriptional profiling of embryonic and adult stem cells" and "a stem cell molecular signature". *Science (New York, N.Y.)*, **302**, 393; author reply 393.

Gao, Z., Cox, J.L., Gilmore, J.M., Ormsbee, B.D., Mallanna, S.K., Washburn, M.P., and Rizzino, A. (2012). Determination of protein interactome of transcription factor Sox2 in embryonic stem cells engineered for inducible expression of four reprogramming factors. The Journal of biological chemistry, **287**, 11384-11397.

Gaspar, J.A., Doss, M.X., Winkler, J., Wagh, V., Hescheler, J., Kolde, R., Vilo, J., Schulz, H. and Sachinidis, A. (2012) Gene expression signatures defining fundamental biological processes in pluripotent, early, and late differentiated embryonic stem cells. *Stem cells and development*, **21**, 2471-2484.

Gautier, L., Cope, L., Bolstad, B. M., Irizarry, R. A. (2004) Affy-analysis of Affymetrix GeneChip data at the probe level. Bioinformatics, **20**, 307-15.

Han, J., Yuan, P., Yang, H., Zhang, J., Soh, B.S., Li, P., Lim, S.L., Cao, S., Tay, J., Orlov, Y.L.*, et al.* (2010). Tbx3 improves the germ-line competency of induced pluripotent stem cells. Nature**, 463**, 1096-1100.

Hansson, J., Rafiee, M.R., Reiland, S., Polo, J.M., Gehring, J., Okawa, S., Huber, W., Hochedlinger, K. and Krijgsveld, J. (2012) Highly coordinated proteome dynamics during reprogramming of somatic cells to pluripotency. *Cell reports*, **2**, 1579-1592.

Hsu, S.D., Lin, F.M., Wu, W.Y., Liang, C., Huang, W.C., Chan, W.L., Tsai, W.T., Chen, G.Z., Lee, C.J., Chiu, C.M.*, et al.* (2011). miRTarBase: a database curates experimentally validated microRNA-target interactions. Nucleic acids research, **39**, D163-169.

Hu, G., Kim, J., Xu, Q., Leng, Y., Orkin, S.H. and Elledge, S.J. (2009) A genome-wide RNAi screen identifies a new transcriptional module required for self-renewal. *Genes & development*, **23**, 837-848.

Isserlin, R., El-Badrawi, R.A., and Bader, G.D. (2011). The Biomolecular Interaction Network Database in PSI-MI 2.5. Database : the journal of biological databases and curation *2011*, baq037.

Ivanova, N.B., Dimos, J.T., Schaniel, C., Hackney, J.A., Moore, K.A. and Lemischka, I.R. (2002) A stem cell molecular signature. *Science (New York, N.Y.)*, **298**, 601-604.

Kerrien, S., Aranda, B., Breuza, L., Bridge, A., Broackes-Carter, F., Chen, C., Duesbury, M., Dumousseau, M., Feuermann, M., Hinz, U.*, et al.* (2012). The IntAct molecular interaction database in 2012. Nucleic acids research, **40**, D841-846.

Keshava Prasad, T.S., Goel, R., Kandasamy, K., Keerthikumar, S., Kumar, S., Mathivanan, S., Telikicherla, D., Raju, R., Shafreen, B., Venugopal, A.*, et al.* (2009). Human Protein Reference Database--2009 update. Nucleic acids research, **37**, D767-772.

Kim, J., Chu, J., Shen, X., Wang, J., and Orkin, S.H. (2008). An extended transcriptional network for pluripotency of embryonic stem cells. Cell, **132**, 1049-1061.

Kim, J., Woo, A.J., Chu, J., Snow, J.W., Fujiwara, Y., Kim, C.G., Cantor, A.B., and Orkin, S.H. (2010). A Myc network accounts for similarities between embryonic stem and cancer cell transcription programs. Cell, **143**, 313-324.

Liang, J., Wan, M., Zhang, Y., Gu, P., Xin, H., Jung, S.Y., Qin, J., Wong, J., Cooney, A.J., Liu, D., Songyang, Z. (2008). Nanog and Oct4 associate with unique transcriptional repression complexes in embryonic stem cells. Nat Cell Biol, **10**, 731-739.

Liu, X., Huang, J., Chen, T., Wang, Y., Xin, S., Li, J., Pei, G., and Kang, J. (2008). Yamanaka factors critically regulate the developmental signaling network in mouse embryonic stem cells. Cell research, **18**, 1177-1189.

Lehner, B., and Fraser, A.G. (2004). A first-draft human protein-interaction map. Genome biology, **5**, R63.

Loh, Y.H., Wu, Q., Chew, J.L., Vega, V.B., Zhang, W., Chen, X., Bourque, G., George, J., Leong, B., Liu, J.*, et al.* (2006). The Oct4 and Nanog transcription network regulates pluripotency in mouse embryonic stem cells. Nature genetics, **38**, 431-440.

Lopes, C.T., Franz, M., Kazi, F., Donaldson, S.L., Morris, Q. and Bader, G.D. (2010) Cytoscape Web: an interactive web-based network browser. *Bioinformatics (Oxford, England)*, **26**, 2347-2348.

Marson, A., Levine, S.S., Cole, M.F., Frampton, G.M., Brambrink, T., Johnstone, S., Guenther, M.G., Johnston, W.K., Wernig, M., Newman, J.*, et al.* (2008). Connecting microRNA genes to the core transcriptional regulatory circuitry of embryonic stem cells. Cell, ***134***, 521-533.

Mathur, D., Danford, T.W., Boyer, L.A., Young, R.A., Gifford, D.K., and Jaenisch, R. (2008). Analysis of the mouse embryonic stem cell regulatory networks obtained by ChIP-chip and ChIP-PET. Genome biology, **9**, R126.

Matys, V., Kel-Margoulis, O.V., Fricke, E., Liebich, I., Land, S., Barre-Dirrie, A., Reuter, I., Chekmenev, D., Krull, M., Hornischer, K.*, et al.* (2006). TRANSFAC and its module TRANSCompel: transcriptional gene regulation in eukaryotes. Nucleic acids research***, 34***, D108-110.

Nitzsche, A., Paszkowski-Rogacz, M., Matarese, F., Janssen-Megens, E.M., Hubner, N.C., Schulz, H., de Vries, I., Ding, L., Huebner, N., Mann, M.*, et al.* (2011). RAD21 cooperates with pluripotency transcription factors in the maintenance of embryonic stem cell identity. PloS one, **6**, e19470.

Palmer, N.P., Schmid, P.R., Berger, B. and Kohane, I.S. (2012) A gene expression profile of stem cell pluripotentiality and differentiation is conserved across diverse solid and hematopoietic cancers. *Genome biology*, **13**, R71.

Pardo, M., Lang, B., Yu, L., Prosser, H., Bradley, A., Babu, M.M., and Choudhary, J. (2010). An expanded Oct4 interaction network: implications for stem cell biology, development, and disease. Cell stem cell, **6,** 382-395.

Persico, M., Ceol, A., Gavrila, C., Hoffmann, R., Florio, A., and Cesareni, G. (2005). HomoMINT: an inferred human network based on orthology mapping of protein interactions discovered in model organisms. BMC bioinformatics, **6** *Suppl 4*, S21.

Ramani, A.K., Bunescu, R.C., Mooney, R.J., and Marcotte, E.M. (2005). Consolidating the set of known human protein-protein interactions in preparation for large-scale mapping of the human interactome. Genome biology, ***6*,** R40.

Rual, J.F., Venkatesan, K., Hao, T., Hirozane-Kishikawa, T., Dricot, A., Li, N., Berriz, G.F., Gibbons, F.D., Dreze, M., Ayivi-Guedehoussou, N.*, et al.* (2005). Towards a proteome-scale map of the human protein-protein interaction network. Nature, **437**, 1173-1178.

Ramalho-Santos, M., Yoon, S., Matsuzaki, Y., Mulligan, R.C. and Melton, D.A. (2002) "Stemness": transcriptional profiling of embryonic and adult stem cells. *Science (New York, N.Y.)*, **298**, 597-600.

Salwinski, L., Miller, C.S., Smith, A.J., Pettit, F.K., Bowie, J.U., and Eisenberg, D. (2004). The Database of Interacting Proteins: 2004 update. Nucleic acids research, **32**, D449-451.

Stelzl, U., Worm, U., Lalowski, M., Haenig, C., Brembeck, F.H., Goehler, H., Stroedicke, M., Zenkner, M., Schoenherr, A., Koeppen, S.*, et al.* (2005). A human protein-protein interaction network: a resource for annotating the proteome. Cell, **122**, 957-968.

Tam, W.L., Lim, C.Y., Han, J., Zhang, J., Ang, Y.S., Ng, H.H., Yang, H., and Lim, B. (2008). T-cell factor 3 regulates embryonic stem cell pluripotency and self-renewal by the transcriptional control of multiple lineage pathways. Stem Cells, **26**, 2019-2031.

Uosaki, H., Fukushima, H., Takeuchi, A., Matsuoka, S., Nakatsuji, N., Yamanaka, S., and Yamashita, J.K. (2011). Efficient and scalable purification of cardiomyocytes from human embryonic and induced pluripotent stem cells by VCAM1 surface expression. PloS one**, 6**, e23657.

van den Berg, D.L., Snoek, T., Mullin, N.P., Yates, A., Bezstarosti, K., Demmers, J., Chambers, I., and Poot, R.A. (2010). An Oct4-centered protein interaction network in embryonic stem cells. Cell stem cell, **6,** 369-381.

Wang, J., Rao, S., Chu, J., Shen, X., Levasseur, D.N., Theunissen, T.W., and Orkin, S.H. (2006). A protein interaction network for pluripotency of embryonic stem cells. Nature, **444**, 364-368.

Wang, C.H., Ma, N., Lin, Y.T., Wu, C.C., Hsiao, M., Lu, F.L., Yu, C.C., Chen, S.Y. and Lu, J. (2012) A shRNA functional screen reveals Nme6 and Nme7 are crucial for embryonic stem cell renewal. *Stem cells (Dayton, Ohio)*, **30**, 2199-2211.
